# Optimierung der Risiko- und Krisenkommunikation von Regierungen, Behörden und Organisationen der Gesundheitssicherung – Herausforderungen in lang anhaltenden Krisen am Beispiel der COVID-19-Pandemie

**DOI:** 10.1007/s00103-023-03708-1

**Published:** 2023-06-06

**Authors:** Annett Schulze, Fabian Brand, Dinah Kristin Leschzyk, Michael Beuthner, Alena Biegert, Udo Bomnüter, Bettina Boy, Hans-Jürgen Bucher, Robert Frau, Marvin Hubig, Martin Löffelholz, Johanne Mayer, Carolyn Pliquet, Johanna Radechovsky, Kathrin Schleicher, Kirsten Ulbrich

**Affiliations:** 1 Abteilung Risikokommunikation, Bundesinstitut für Risikobewertung (BfR), Max-Dohrn-Str. 8–10, 10589 Berlin, Deutschland; 2 SRH Berlin School of Applied Sciences, Berlin School of Popular Arts, Berlin, Deutschland; 3 Macromedia University of Applied Sciences, Campus Berlin, Berlin, Deutschland; 4 Karlsruher Institut für Technologie (KIT), Karlsruhe, Deutschland; 5 Europa-Universität Viadrina, Frankfurt (Oder), Deutschland; 6 Technische Universität Ilmenau, Ilmenau, Deutschland

**Keywords:** Multimodalität, Krisendispositiv, Datenvisualisierung, Rechtssicherheit, Fehlinformation, Multimodality, Crisis dispositive, Data visualization, Legal certainty, Disinformation

## Abstract

Die COVID-19-Pandemie illustriert die besondere Bedeutung von Risiko- und Krisenkommunikation. Behörden und Politik stehen vor der Herausforderung, in einer dynamischen Lage mit einer Vielzahl von Daten umzugehen, diese zu überprüfen und zielgruppengerecht zu kommunizieren. Verständliche und eindeutige Informationen zu Risiken und Handlungsoptionen tragen maßgeblich zu einer Steigerung der objektiven und subjektiven Sicherheit der Bevölkerung bei. Es besteht daher ein großer Bedarf, die Erfahrungen aus der Pandemie in die Optimierung der Risiko- und Krisenkommunikation einfließen zu lassen.

Die Digitalisierung ermöglicht multimodale Arrangements – also die Kombination aus Text, Abbildungen, Grafik, Icons und z. T. Bewegtbilder, Animationen und Ton. Diese spielen auch in der digitalen Risiko- und Krisenkommunikation eine zunehmend wichtigere Rolle. Von Interesse ist, inwiefern das kommunikative Zusammenspiel von Behörden, Medien und weiteren Öffentlichkeitsakteur/-innen in Vorbereitung auf und zur Bewältigung von Krisen angesichts einer komplexen Öffentlichkeit mit Hilfe zielgruppenspezifischer Kommunikation verbessert und wie Rechtssicherheit für die behördliche und mediale Praxis gewährleistet werden kann. Dementsprechend verfolgt der Beitrag 3 Ziele:

1. Er beschreibt die Herausforderungen, vor denen Behörden und mediale Akteur/-innen in der Pandemiekommunikation stehen.

2. Er zeigt, welche Rolle multimodale Arrangements spielen und welcher Forschungsperspektiven es bedarf, um die Komplexität des kommunikativen Krisenhandelns im föderalen System zu erfassen.

3. Er begründet, wie ein interdisziplinärer Forschungsverbund aus Medien‑, Kommunikations- und Rechtswissenschaft Erkenntnisse zum evidenzbasierten Einsatz multimodaler Kommunikation gewinnen kann.

## Abstract (en)

The COVID-19 pandemic demonstrates the great importance of risk and crisis communication. In a dynamic situation, authorities and policymakers face the challenge of dealing with a large amount of data, reviewing it and communicating it in a way that is appropriate for diverse target groups. Comprehensible and unambiguous information on risks and options for action make a significant contribution to the objective and subjective safety of the population. Hence, there is a great need to use the experience gained from the pandemic to optimize risk and crisis communication.

Digitalization enables multimodal arrangements – that is, the combination of text, graphics, icons, images, animations and sound. These arrangements play an increasingly important role in risk and crisis communication. It is of interest to what extent the communicative interaction of authorities, media and other public actors in crisis preparation and management in view of a complex public can be improved with the help of target group-specific communication and how legal certainty can be ensured for official and media practice. Accordingly, the article pursues three objectives:

1. It describes the challenges faced by authorities and media actors in pandemic communication.

2. It shows the role of multimodal arrangements as well as the necessary research perspectives to grasp the complexity of communicative crisis management in the federal system.

3. It provides a rationale for how an interdisciplinary research network from the fields of media, communication and law can gain insights into the evidence-based use of multimodal communication.

## Einleitung

In Krisen wie der COVID-19-Pandemie sind staatliche Institutionen mit einer komplexen und dynamischen Situation konfrontiert, die von hoher Unsicherheit und potenziell dramatischen Konsequenzen für die Bevölkerung geprägt ist [1]. Daher versuchen Regierungen, Behörden und Organisationen der Gesundheitssicherung die negativen Folgen so gering wie möglich zu halten – nicht zuletzt durch kommunikative Maßnahmen [2]. So kommt Regierungen bei der Verbreitung akkurater, zuverlässiger und schneller Informationen eine große Verantwortung zu [3], wobei die Risiko- und Krisenkommunikation in Demokratien, insbesondere in föderalen Systemen, vielstimmig und mediatisiert ist [4, 5].

Der Diskurs zur Bewältigung der COVID-19-Pandemie entfaltet sich in einer Öffentlichkeit, die nicht mehr nur massenmedial geprägt, sondern durch den „digitalen Wandel der Wissensordnung“ [6] und der Herausbildung eines „hybriden Mediensystems“ [7] instabiler geworden ist. Grund dafür sind die Erweiterung der kommunikativen Partizipations- und Einflusspotenziale eines vormals auf die Rezeption journalistischer wie politischer Informationsangebote beschränkten Publikums und das zunehmende Erodieren der Möglichkeiten klassischer Kontrollinstanzen zur Steuerung der öffentlichen Kommunikation. Gleichzeitig ist die Öffentlichkeit durch eine vermehrte Multimodalisierung medialer Kommunikation [8, 9] gekennzeichnet.

Polarisierungen in öffentlichen Debatten um Wahrheit und die Gültigkeit von Wissen werden Teil des kommunikativen Alltags und können dabei auch in den sozialen Medien (Social Media) bedeutsam werden. Die Diversität und Eigenlogik der Plattformen beeinflussen die Formate und damit auch die Kommunikationsbeiträge, da Beiträge an die verschiedenen Zielgruppen und Kommunikationskanäle angepasst werden müssen [10]; fehlende Verantwortlichkeiten für den veröffentlichten Inhalt erschweren den Umgang mit Informationen [11].

Bislang liegen weder Befunde zum Zusammenspiel von neuen Öffentlichkeitsakteur/-innen (wie etwa Influencer/-innen), Medien sowie Behörden und Organisationen der Gesundheitssicherung vor noch existiert eine empirisch gesättigte Konzeptualisierung des Social-Media-Einflusses im Krisenkontext [12]. Hier setzt der Forschungsverbund MIRKKOMM (Optimierung der Risiko- und Krisenkommunikation von Regierungen, Behörden und Organisationen der Gesundheitssicherung)^1^ an. Im Erkenntnisinteresse liegen dabei insbesondere die Risiko- und Krisenkommunikation in sich ändernden Lagen, das jeweils aktuelle Wissen und Handlungsempfehlungen. Das Projekt verbindet kommunikative Fragestellungen zur Effektivität multimodaler Krisen- und Risikokommunikation mit Usability-Aspekten in Mensch-Maschine-Interaktionen sowie mit Aspekten der Rechtssicherheit von Kommunikation.

Im Folgenden werden erstens die konkreten pandemiespezifischen Herausforderungen von Behörden und Organisationen der Gesundheitssicherung und medialen Akteur/-innen sowie zweitens die Rolle multimodaler Arrangements in der Risiko- und Krisenkommunikation herausgearbeitet. Dabei werden verschiedene Forschungsperspektiven dargestellt, die es ermöglichen, die Komplexität des kommunikativen Krisenhandelns zu erfassen. Im dritten Schritt werden die daraus erforderlichen Forschungsansätze und -perspektiven abgeleitet. Erläutert wird dabei das Forschungsvorhaben des interdisziplinären Forschungsverbunds MIRKKOMM, das Erkenntnisse zum evidenzbasierten Einsatz multimodaler Kommunikation durch staatliche und mediale Akteur/-innen generiert.

Ziel des Beitrags ist es, Optimierungspotenziale einer multimodalen Krisenkommunikation und deren Umsetzungsmöglichkeiten aufzuzeigen.

## Herausforderungen behördlicher und medialer Risiko- und Krisenkommunikation

In Pandemien sind zentrale kommunikative Aufgaben zu bewältigen [13]: das *Informieren* der Bevölkerung, das *Korrigieren* von überholter und unzutreffender Information und das *Mobilisieren* für und *Erklären *von Schutzmaßnahmen. Damit verbunden sind unterschiedliche Bedürfnisse und Ansprüche an Krisenkommunikation. Anhand der COVID-19-Pandemie werden diese Aufgaben exemplarisch dargestellt.

### Strukturelle Herausforderungen

Die COVID-19-Pandemie ist zu einer „chronischen“ Krise geworden [14]: Die anhaltende Pandemie erfordert eine kontinuierliche Aufmerksamkeit für die Schwankungen, denen die Motivation, die Handlungsbereitschaft oder die psychosozialen Empfindungen der Betroffenen unterworfen sind. Zudem ändern sich die Charakteristika der Krise selbst sowie die Handlungsoptionen und Wissensbestände. Diese Konstellationen und Dynamiken manifestieren sich in verschiedenen Phasen. Das Robert Koch-Institut (RKI) skizzierte retrospektiv den pandemischen Verlauf u. a. anhand epidemiologischer Daten [15]. So lassen sich neben der Ausbreitungsphase, den Lockerungen und dem Sommerplateau im ersten Jahr der Pandemie (2020) verschiedene Ansteckungswellen ausmachen, in denen unterschiedliche politische und kommunikative Maßnahmen zum Schutz der Bevölkerung ergriffen wurden.

Diese Maßnahmen beruhten u. a. auf einer wissenschaftsbasierten Politikberatung. Ihre Umsetzung fand a) durch öffentliche Einrichtungen statt, deren knappe Personalausstattung und administrative Strukturen immer wieder herausforderten [16], und b) in einer föderalen und mediatisierten Gesellschaft, in der zugleich und divers kommuniziert wurde [4, 5]. In den zuständigen Organisationseinheiten fehlten krisentaugliche Strukturen für die interne und externe Kommunikation von Behörden [5, 17], d. h., es mangelt mitunter an Expertise verhaltenswissenschaftlich-basierter Kommunikation, deren Grundlage jeweils aktuelle Datenerhebungen bilden. Darüber hinaus spielt der Rechtsrahmen eine entscheidende Rolle für die Risiko- und Krisenkommunikation. Denn auch eine Gesellschaft im Krisenmodus bleibt rechtlich konstituiert. Staatliche Kommunikation ist bisher nur rudimentär über verfassungsrechtliche und verfassungsgerichtliche Vorgaben geregelt.

### Unsichere Datenlage und Fehlinformationen

Bei SARS-CoV‑2 handelt es sich um einen Erreger, über den anfänglich bspw. Details zu den Übertragungswegen nicht bekannt waren. Eine genaue Beschreibung der Erkrankung konnte erst nach und nach vervollständigt werden.^2^ Gerade im ersten Jahr der Pandemie mussten verlässliche Meldewege an die zuständigen Behörden etabliert und neue Daten u. a. zu Todesfällen und für die Entwicklung wirksamer Impfungen und Medikamente erhoben werden [15, 16, 18–20]. Zudem gab es Unsicherheiten in Bezug auf die Wirksamkeit und Verfügbarkeit unterschiedlicher Maskenarten [17, 21].

Diese unsichere Datenlage zu kommunizieren, ist eine weitere Herausforderung, vor allem weil zunehmend wissenschaftliches und politisches Wissen über soziale Medien bezogen wird [22]. In sozialen Medien wiederum lassen sich Fehlinformationen leicht und schnell verbreiten; das zeigt sich auch in der COVID-19-Pandemie [23, 24]. Dabei können Fehlinformationen definiert werden als Inhalte, die nicht auf Fakten basieren und mutwillig oder unwissentlich weitergegeben werden [25].

So agieren staatliche Akteur/-innen und journalistische Medien in komplexen, multidirektionalen Kommunikationsprozessen, die durch andere Öffentlichkeitsakteur/-innen auf Social-Media-Plattformen wie „Influencer/-innen“ mitgeprägt werden (siehe auch der Abschnitt zur Kommunikator/-innenperspektive). Diese beteiligen sich an der Kommunikation über eine Krise unmittelbar, haben mitunter hohe Reichweiten und nutzen das Internet, um Follower- oder Klickzahlen zu generieren und Aufmerksamkeit für ihre Positionen zu erzeugen [26].

Die Europäische Kommission wies darauf hin, dass Gerüchte oder Falschinformationen staatliche Anstrengungen unterminieren, also z. B. dazu führen, dass „Menschen Gesundheitshinweise offizieller Stellen ignorieren und durch ihr Verhalten Risiken eingehen“ [27]. Politische und wissenschaftliche Institutionen bekämpfen folglich – bspw. über Richtigstellungen – „nicht nur eine Epidemie, sondern eine Infodemie“ [28], d. h. eine übermäßige Menge an Informationen, die sich „schneller und leichter verbreiten als dieses Virus“ [28]. Zur Wirksamkeit solcher immer wieder geforderten Debunking^3^-Versuche [29] zeigen verschiedene Forschungsdesigns unterschiedliche Ergebnisse. Abhängig von der Zielgruppe und deren Einstellung können sowohl Verstärkereffekte als auch Abschwächungen mit Blick auf die Glaubwürdigkeit von Desinformationen eintreten [30].

### Datengetriebene Kommunikation und Literacies

Je länger die COVID-19-Pandemie dauerte, desto stärker war die Pandemiekommunikation durch quantifizierbare Daten geprägt: Epidemiologische Daten wurden z. B. in Dashboards und Apps visualisiert und öffentlich einsehbar [31]. Das erste Dashboard, das bereits Ende Januar 2020 vorhandene Daten in Echtzeit veröffentlichte und von zahlreichen Medien weltweit referenziert wurde, war von der Johns Hopkins University im US-Bundesstaat Maryland [24, 32].

Verantwortliche im Krisenmanagement leiteten aus den epidemiologischen Daten und aus Beratungen durch Gremien mit unterschiedlicher wissenschaftlicher Expertise Maßnahmen ab, nutzten Umfragen, um Prognosen zu erstellen und eine aktuelle, zielgruppenspezifische Krisenkommunikation zu entwickeln. Allein die Rezeption digitaler Daten führt jedoch nicht zu einem entsprechenden Verständnis. Es bedarf vielmehr der Datenkompetenz („data literacy“), sowohl in der Bevölkerung als auch im politisch-administrativen Bereich [33, 34]. Die Datenkompetenz hängt eng mit der Gesundheitskompetenz („health literacy“) zusammen. Verfügen Menschen über eine hohe Gesundheitskompetenz, sind sie in der Lage, „gesundheitsrelevante Informationen erschließen, verstehen, beurteilen und konstruktiv zur Entscheidungsfindung bei Gesundheitsfragen nutzen zu können“ [35].

In Deutschland besitzen laut des zweiten Health Literacy Survey Germany allerdings nur rund 40 % der Bevölkerung eine hohe Gesundheitskompetenz [35]. Zudem müssen (fehlende oder wenig ausgeprägte) Kompetenzen im Umgang mit Risiken („risk literacy“^4^) berücksichtigt werden.

### Aufbereitung der Informationen

In der Umsetzung von Kommunikationsformaten und -botschaften sind diverse Parameter für unterschiedliche Zielgruppen zu berücksichtigen, die ein theorie- und evidenzbasiertes Vorgehen in der Entwicklung der entsprechenden Kommunikation in Gesundheitskrisen erfordern [26, 36]. Das bedeutet, dass die Inhalte textlich, auditiv und visuell aufbereitet werden müssen. Online verfügbare wissenschaftliche Stellungnahmen, Positionspapiere etc. waren bislang häufig textlastig gestaltet [37]. So müssen Kommunikator/-innen antizipieren, wer auf welche Information wie reagiert, denn die Bedeutung bzw. der kohärente Gesamtsinn von Kommunikaten entsteht erst in der Interaktion der Rezipient/-innen mit dem Material [38]. Die Konstruktion von Bedeutung muss demnach als interaktiver Prozess verstanden werden [38].

## Multimodale Arrangements in der Risiko- und Krisenkommunikation

### Theorie und Begriff der Multimodalität

Im Verlauf der Mediengeschichte wurde Kommunikation zunehmend durch den simultanen Einsatz verschiedener Kommunikationsmodi realisiert, wie gesprochene oder geschriebene Sprache, Grafiken, Bewegtbilder, Farben, Intonation, Design oder Typografie, die in multimodalen Arrangements organisiert sind [39]. Diese sind am reichhaltigsten in den digitalen Medien ausgeprägt [9].

Multimodalität im Kontext der Risiko- und Krisenkommunikation von Behörden und Organisationen der Gesundheitssicherung, Medien und Social-Media-Akteur/-innen zu erforschen, ermöglicht ein Verständnis für die Bedeutung einzelner Modi sowie für die Gesamtbedeutung des Arrangements [40, 41] und gibt Aufschluss darüber, welche Bedeutungspotenziale Informationen in Warnungen oder Handlungsempfehlungen beinhalten und wie diese interpretiert bzw. verstanden werden können [10].

Der Begriff des Verständnisses erfordert, zwei Dimensionen zu berücksichtigen: Zum einen ist das, was verstanden wird, abhängig von Variablen wie Vorwissen, Erfahrungen oder sozialen Normen. Zum anderen geht der kohärente Gesamtsinn, den Rezipient/-innen von einem Medienangebot entwickeln, über das Verständnis der einzelnen Modi hinaus: Er entsteht multiplikatorisch [38]. Es macht bspw. einen Unterschied, ob eine Institution textlastig oder über Videos kommuniziert, die eine höhere modale Dichte, sprich Bild, Text, Ton etc., aufweisen. Ein hohes Maß an unterschiedlichen Darstellungsmodi in einem Kommunikat ist allerdings nicht automatisch mit einer höheren Verständlichkeit des Kommunikationsangebotes gleichzusetzen. Lassen die genutzten Modi unterschiedliche Botschaften zu oder überfordern in ihrer multimodalen Orchestrierung, so konfligieren diese mit dem Anspruch an Eindeutigkeit und Kohärenz. Gleichzeitig kann ein adäquates Arrangement von bspw. Ton, Text und Layout die Wissensvermittlung stützen [38].

Zum Einsatz multimodaler Kommunikation in der Risiko- und Krisenkommunikation liegen jedoch bislang kaum Befunde vor, obgleich Bürger/-innen in Notlagen zunehmend visuelle und auditive Inhalte über soziale Medien teilen [42–44]; tendenziell werden multimodale Kommunikationsarrangements eher verbreitet [45, 46]. Generell wächst die Relevanz von Analysen zu den visuellen Modi der Bedeutungskonstruktion von Ereignissen [43] oder zum strategischen Management visueller Kommunikation [47]. Es besteht zudem die These, dass kommunikative Praktiken, auch in der Risiko- und Krisenkommunikation, nur durch multimodale Analysen tiefenscharf zu erschließen seien [43–45, 47].

Ziel des Forschungsverbunds MIRKKOMM ist es daher, Daten zu einem evidenzbasierten Einsatz multimodaler Kommunikation durch staatliche und mediale Akteur/-innen zu liefern, um so – auch mit Blick auf begrenzte Ressourcen – sinnvolle Optionen für die Risiko- und Krisenkommunikation aufzuzeigen.

### Beispielkommunikate der COVID-19-Pandemie: Dashboards und Richtigstellungen

Im Kontext der COVID-19-Pandemie setzen verschiedene Behörden und Organisationen der Gesundheitssicherung, Medien und Social-Media-Akteur/-innen unterschiedliche Kommunikationsformate ein. An Beispielen dieser Kommunikationsformate lassen sich die Herausforderungen und Dilemmata multimodaler Risiko- und Krisenkommunikation prägnant darstellen.

**Figure 1:**
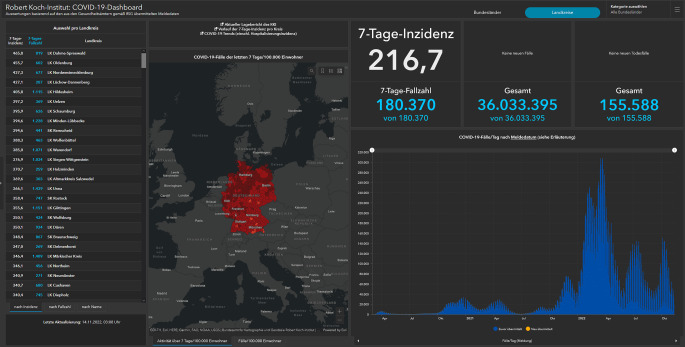

Dashboards stehen in dieser Pandemie wie kein anderes Format für die Herausforderungen in der Visualisierung wissenschaftlicher Daten. Institutionen wie das Bundesministerium für Gesundheit (BMG; [48]), das RKI (Abb. 1; [49]), einzelne Bundesländer [50] oder Medien [51] nutzen Dashboards, um ihre Zielgruppen über den Impfstand, aktuelle Fallzahlen, Hospitalisierungen, Intensivbettenbelegung und Todesfälle unter Einsatz verschiedener Visualisierungsformen zu informieren.

Aus Perspektive der Multimodalitätsforschung sind diese Dashboards von Interesse, da in einem komplexen visuellen Arrangement versucht wird, heterogene Daten überblicksartig darzustellen. Dabei werden unterschiedliche quantitative, geografische oder zeitliche Daten über verschiedene Darstellungsformen – wie Karten oder Verlaufsdiagramme – abgebildet, die von Rezipient/-innen eine nichtlineare, selbstgesteuerte Nutzung erfordern. Damit werden Rezipient/-innen vor die Herausforderung gestellt, ein Verständnis für die räumliche Struktur verschiedener Elemente eines simultan präsentierten Kommunikationsangebotes zu gewinnen. Für die Multimodalitätsforschung werden hierbei nicht nur Fragen nach einem sinnvollen – also einem Verständlichkeit, Nachvollziehbarkeit und Vertrauen fördernden – Arrangement verschiedener Kommunikationsmodi relevant, sondern auch nach der Art und Weise, wie solche Formate möglichst effektiv Überzeugungskraft entfalten können [52].

**Figure 2:**
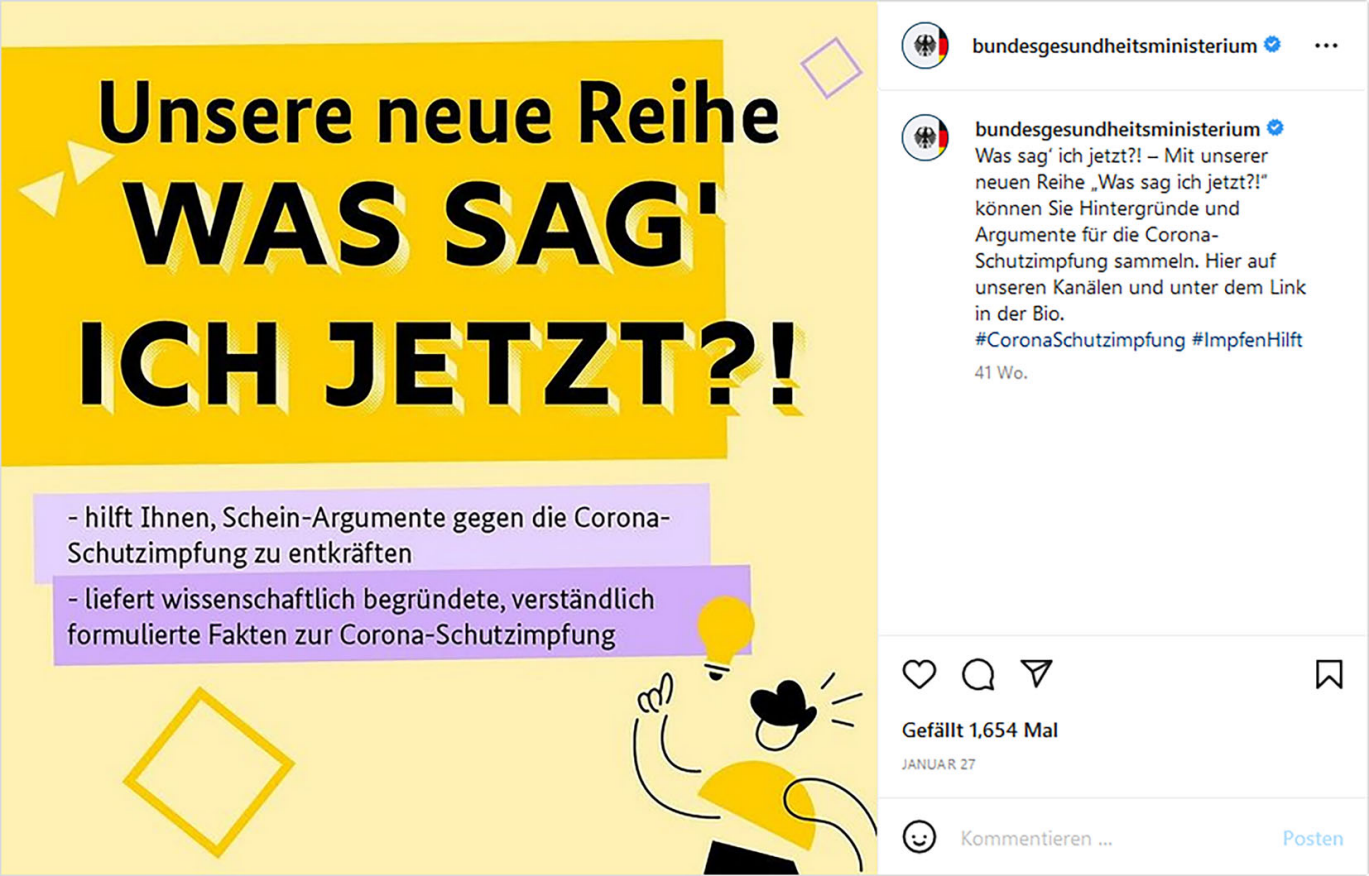

Ein weiteres Kommunikationsformat, das in der Pandemie bedeutsam wurde, sind Richtigstellungen – als Elemente von breiter angelegten Kommunikationskampagnen oder aber als genuin eigenes Kampagnenformat. Diese wurden u. a. vom BMG unter dem Motto „Was sag’ ich jetzt“ im Rahmen einer Impfkampagne auf Social-Media-Plattformen publiziert (Abb. 2). Dabei sollen „Schein-Argumente gegen die Corona-Schutzimpfung“ entkräftet und „wissenschaftlich begründete, verständlich formulierte Fakten zur Corona-Schutzimpfung“ geliefert werden [53].

Studien belegen die Konsequenzen von Fehlinformationen, darunter die Tatsache, dass Personen mit einem hohen Vertrauen in wissenschaftliche Fakten [54] oder einer häufigen Nutzung sozialer Medien [55] besonders anfällig für pseudowissenschaftliche Fehlinformationen sind. So scheinen Richtigstellungen nötig. Ihre Effektivität wird jedoch kontrovers diskutiert [30, 56, 57]. Aus Perspektive der Multimodalitätsforschung ist die Effektivität von Richtigstellungen noch weitestgehend unbekannt. Einen ersten Schritt in diese Richtung haben Hameleers et al. [58] unternommen und untersucht, inwieweit sich Multimodalität auf die Glaubwürdigkeit von Fehlinformationen auswirkt.

## Forschungsanalytische Perspektiven des Forschungsverbunds MIRKKOMM

Im Folgenden werden die vier im Forschungsverbund zentralen Analyseperspektiven skizziert und weitere Desiderata aufgezeigt.

### Kommunikator/-innenperspektive: intra- und interbehördliche Kommunikation

Bislang sind staatliche Kommunikator/-innen in Krisen von der Forschung vergleichsweise wenig untersucht worden [59]. Erste Analysen [60] zeigen die Relevanz von Form und Verbreitung von Informationen während der COVID-19-Pandemie. Eine immer wichtigere Rolle spielen demnach die sozialen Medien [3]; sie ermöglichen, Botschaften schnell und effizient zu verbreiten, auf verschiedene Zielgruppen zuzuschneiden [61] und in Echtzeit zu aktualisieren [62]. Die kommunikative Umsetzung staatlicher Strategien zur Krisenbewältigung gerade in grenzüberschreitenden Lagen wie einer Pandemie und darauf aufbauende Lerneffekte können durch eine unangemessene Auswahl und fehlende multimodale Aufbereitung von Informationen behindert werden [63, 64]. Die Perspektive der staatlichen Akteur/-innen berücksichtigt daher auch die Kommunikation mit digitalen und partizipativen Medien.

Damit ihre Empfehlungen akzeptiert, das Verhalten der Bevölkerung angepasst [65] und Krisensituationen effektiv bewältigt werden können [66], soll behördliche Krisenkommunikation vor allem Sicherheit und Vertrauen erzeugen [67–70]. Erfolgreiche staatliche Krisenkommunikation setzt voraus, dass geeignete Kommunikationsstrategien ausgewählt, deren Wirkung richtig eingeschätzt, die Beziehungen zu den Zielgruppen angemessen und differenziert gestaltet [59, 62, 70], eine Kohärenz der kommunikativen Maßnahmen auf verschiedenen staatlichen Ebenen geschaffen und die Selektivität der Medienberichterstattung berücksichtigt werden [71].

Um wirksam zu kommunizieren, sollen staatliche Akteur/-innen in ihrer Krisenkommunikation proaktiv gegen Fehlinformationen vorgehen [61]. Ein zweiseitiger Kommunikationsprozess ist für die Umsetzung der kommunizierten Maßnahmen, die Geschwindigkeit und den Umfang der staatlichen Reaktion von entscheidender Bedeutung [61]. In Krisen müssen akkurate, motivierende Verhaltensinstruktionen gegeben werden [17], um die Bevölkerung vor Schäden zu bewahren [72], ohne jedoch Ängste und ggf. Panik auszulösen [73].

Inwieweit diese Ansprüche aus Sicht der staatlichen Institutionen im Rahmen der COVID-19-Pandemie erfüllt werden und ob frühere Defizite der Risiko- und Krisenkommunikation staatlicher Akteur/-innen [74] überwunden wurden, wird im Rahmen dieses Teilvorhabens durch die Analyse der intra- und interbehördlichen Kommunikation über Interviews, eine Repräsentativbefragung und eine Dokumentenanalyse geprüft werden.

### Kommunikator/-innenperspektive: Kommunikation etablierter Medien und Social-Media-Akteur/-innen

Journalist/-innen und Social-Media-Akteur/-innen sind entscheidende Intermediäre in der Kommunikation zwischen Behörden/Organisationen der Gesundheitssicherung und der Gesellschaft, weil sie notwendige Transfer- und Transformationsleistungen für ihre Zielgruppen erbringen. Sie fördern Meinungsbildung, schaffen Orientierung, aber auch Distanz oder Ablehnung.

In der COVID-19-Pandemie haben Medienhäuser wie der Zeitverlag Daten getrackt und analysiert, grafisch aufbereitet und der Öffentlichkeit zur Verfügung gestellt [75]. Redaktionen sind in den Dialog mit ihren Lesenden getreten, um herauszufinden, wie sie die Datenaufbereitung verständlicher gestalten können. Journalismus in der Pandemie wurde dementsprechend auch zum Daten- und Wissenschaftsjournalismus und erforderte Fähigkeiten in der Datenrecherche, dem Verstehen, der Einordnung und der Überprüfung von epidemiologischen u. a. Erkenntnissen [76]. Social-Media-Akteur/-innen, die als Influencer/-innen gelten, hatten ebenfalls zentrale Rollen in der Pandemiekommunikation, z. B. als Botschafter in strategischen Kampagnen von Behörden oder als „alternative Influencer“ in der Verbreitung von Fehlinformationen [22].

Ein Teilprojekt beschäftigt sich mit einer systematischen Erfassung der Erwartungen von Journalist/-innen und Social-Media-Akteur/-innen gegenüber dem gelieferten Content der Behörden und Organisationen der Gesundheitssicherung. Analysiert wird, wie Entscheidungs- und Produktionsprozesse der Journalist/-innen und Influencer/-innen die multimodale Gestaltung eigener Beiträge und Botschaften beeinflussen, in denen behördliche Informationen kommentiert, reformuliert oder rekontextualisiert werden. Es werden zudem die Gründe für die multimodale Gestaltung von Botschaften für spezifische Zielgruppen sowie die damit verbundenen kommunikativen Funktionen und Absichten erforscht. Dazu werden jeweils 40 leitfadengestützte Expert/-inneninterviews mit Akteur/-innen aus dem Journalismus und Social Media durchgeführt.

### Produktanalytische Perspektive: Diskurs und Krisendispositiv

Die produktanalytische Perspektive schließt an die Kommunikator/-innenperspektive an und untersucht über eine multimodale Diskursanalyse den produzierten Inhalt von Behörden und Organisationen der Gesundheitssicherung, Medien und Social-Media-Akteur/-innen.

Von Interesse sind erstens die multimodalen Arrangements in den Kommunikationsangeboten. Zweitens interessiert das spezifische Krisendispositiv dieser Pandemie. Als zentrales Konstrukt beschreibt es die Regeln gesellschaftlicher Ordnung, die sich u. a. in administrativen Maßnahmen, in anerkanntem Wissen, in Moralvorstellungen und Ethikrichtlinien verwirklichen [16, 77]. Es zeigt sich darin, wie auf eine Krisensituation reagiert wird, welche Maßnahmen auf politisch-administrativer Ebene wie kommuniziert werden, welche wissenschaftlichen Erkenntnisse von wem in die Öffentlichkeit gebracht werden und was zu welchem Zeitpunkt in der Pandemie ungesagt bleibt [78].

Die produktanalytische Perspektive ermöglicht Erkenntnisse dazu, wie unterschiedliche Akteur/-innen über bewährte und neue Sprecherpositionen, z. B. über den akademischen Grad und die spezifische wissenschaftliche Disziplin (Professur der Virologie), in Krisen über Text‑, Bild- und Grafik-Repertoires interagieren, inwieweit diese Arrangements Kohärenzen, aber auch Widersprüchlichkeiten enthalten und welche Deutungsmuster in Krisennarrativen zum Tragen kommen. Die Untersuchung des Krisendispositivs liefert Antworten darauf, wer in Krisen eine Stimme bekommt und welche Positionen im öffentlichen Diskurs als anerkannt oder (un)wahr konstruiert werden.

Erwartet werden Befunde über a) die gesellschaftlichen Normen, Regeln und Konventionen, die in der Risiko- und Krisenkommunikation das Krisendispositiv strukturieren [79, 80], b) Wissenselemente und Positionen, die gesellschaftlich anerkannt oder aber in Frage gestellt werden, und c) die Potenziale einzelner Modi und die Handlungsoptionen durch multimodale Arrangements.

### Rezeptionsperspektive: Wahrnehmung und Wirkung von Krisen- und Risikokommunikation in der COVID-19-Pandemie

Der Erfolg von Krisen- und Risikokommunikation misst sich an der Rezeption der Kommunikationsangebote aufseiten ihrer Adressat/-innen. Die Kommunikation in einer pandemischen Krise ist einerseits durch eine volatile und zum Teil auch widersprüchliche Informationslage und andererseits durch hohen Handlungsdruck aller Akteur/-innen gekennzeichnet. Dieses Krisendispositiv aus epistemischer Unsicherheit und Handlungsdruck muss die Pandemiekommunikation einkalkulieren. Ihr Erfolg bemisst sich deshalb nicht nur an einer gelungenen Wissensvermittlung, sondern auch an der Förderung von Vertrauen und Akzeptanz gegenüber behördlichen Entscheidungen sowie an einer Mobilisierung der Adressat/-innen zu präventivem Handeln wie der Befolgung von Schutzmaßnahmen [63].

Ob die Kommunikation von Behörden diesen Anforderungen gerecht wird, ist eine empirische Frage, deren Beantwortung eine mehrschichtige Rezeptionsforschung erforderlich macht: Sie muss sowohl kognitive als auch emotionale Effekte messbar machen und zeigen, wie Kommunikationsangebote verstanden werden, wie sie die Risikowahrnehmung der Adressat/-innen beeinflussen, nach welchen Kriterien sie bewertet und welche konkreten Handlungsanforderungen daraus abgeleitet werden.

Da die modale Orchestrierung eines Kommunikationsangebotes sowohl für die Verteilung der Aufmerksamkeit der Rezipient/-innen als auch für deren emotionales Involvement eine entscheidende Rolle spielt, sind konkrete Rezeptionsbefunde im Hinblick auf die Modalitätskriterien die Grundlage für eine medienbasierte pandemische Kommunikationsstrategie [38, 41, 80]. Ausgangspunkt der Teilstudie zur Rezeption ist eine Typologie pandemischer Kommunikationsangebote, die nach Funktionen, multimodalen Mustern, Themen und Akteur/-innen aufgebaut ist. Darüber hinaus werden die medialen Angebote nach ihrer Einbettung in verschiedene Pandemiephasen unterschieden und systematisiert.

Die Rezeptionsstudie umfasst eine Blickaufzeichnung, die die Aufmerksamkeitsverteilung auf verschiedene Modalitäten misst, einen Wissenstest zur Erhebung der Informationsvermittlung und eine Befragung, mit der emotionale Aspekte wie Handlungsbereitschaft, Vertrauen und Einstellungen erfasst werden. Die Verbindung von Produkt- und Rezeptionsanalyse im Rahmen des Teilprojekts ermöglicht es, systematische Zusammenhänge zwischen der multimodalen Gestaltung der Pandemiekommunikation in sozialen Medien und deren Kommunikationserfolg zu erkennen sowie entsprechende Empfehlungen für die Krisenkommunikation abzuleiten.

Ergänzt werden die Studien um die Analyse des für den Zivil- und Katastrophenschutz entwickelten Modularen Warnsystems (MoWaS) sowie dessen Usability. Das bundesweite Warnsystem wurde durch das Bundesamt für Bevölkerungsschutz und Katastrophenhilfe (BBK) beauftragt und von dem Unternehmen mecom Medien-Communikations-Gesellschaft mbH entwickelt. MoWaS erweitert den Erkenntnisgewinn anderer Teilvorhaben im Bereich der behördlichen Warnungen im Kontext der Mensch-Maschine-Interaktion. Untersucht wird qua Leitfadeninterviews u. a., inwiefern das multimodale Warnsystem MoWaS die Erstellung von Warnmeldungen an die Bevölkerung erleichtert.

### Rechtswissenschaftliche Perspektive

Im Rechtsstaat des Grundgesetzes bedarf es aufgrund des Prinzips des Vorbehalts des Gesetzes einer für staatliches Handeln einfachgesetzlichen Rechtsgrundlage, um Eingriffe in subjektiv öffentliche Rechte, zu denen insbesondere die Grundrechte zählen, zu ermöglichen, andernfalls sind sie rechtswidrig. Ob ein Grundrechtseingriff vorliegt, wird im Einzelfall durch eine Schutzbereichsprüfung bestimmt.

Kommunikation und Information, soweit sie nicht zielgerichtet auf Grundrechtsgehalte einwirken, führen im Regelfall nicht zu solchen Grundrechtseingriffen. Ob dem aber tatsächlich so ist, wird in einem juristischen Teilvorhaben untersucht.

Grundrechtseingriffe lassen sich nicht ausschließen: Staatliche Informationstätigkeit kann die Form von Warnungen annehmen und ggf. die Adressat/-innen so stark beeinflussen, dass die Schwelle zum Grundrechtseingriff überschritten wird. Letztlich beeinflusst staatliches Auftreten im Äußerungskontext die Akzeptanz und damit die Legitimität staatlicher Hoheitsgewalt [81] und wird damit aus der Perspektive des Verfassungs- und Verwaltungsrechts relevant, je nachdem, in welcher Form sich staatliche Äußerungen darstellen.

Herausfordernd ist, dass staatliche Informations- und Kommunikationstätigkeit rechtlich nur rudimentär geregelt ist, wie es sich in den fehlenden Gesetzen auf Landes- und Bundesebene darstellt. Die Analyse im Teilvorhaben zeigt, dass kein Bundesland entsprechende Gesetze erlassen hat; es mangelt an einer Regelung auf Bundesebene. Rechtliche Vorgaben für Kommunikation sowohl in ruhigen Zeiten als auch in Krisen müssen daher aus dem Grundgesetz und einigen Spezialgesetzen destilliert werden. Ausgangspunkt hierfür bildet die Rechtsprechung des Bundesverfassungsgerichts (BVerfG), die seit den 1970er-Jahren immer wieder die hoheitlichen Kommunikationsbefugnisse betrifft und in der insbesondere zwei Entscheidungen (E) aus dem Jahr 2002 hervorstechen.

Inhaltlich ging es um zwei Warnungen, welche die Bundesregierung wegen kontaminierten Weins und Jugendsekten herausgegeben hatte (BVerfGE 105, 252; BVerfGE 105, 279). Das BVerfG hat in diesen Entscheidungen herausgearbeitet, dass das Grundgesetz hoheitliche Kommunikation zulässt, wenn sie sachlich, weiterhin politisch neutral und im Rahmen hoheitlicher Kompetenzen bleibt. In den letzten Jahren standen die Äußerungsbefugnisse von Mitgliedern der Bundesregierung und des Bundespräsidenten im Fokus der bundesverfassungsgerichtlichen Rechtsprechung (BVerfGE 136, 323). In diesen Entscheidungen betonte das Gericht die parteipolitische Neutralität, die solche Funktionsträger zu wahren haben.

Beide Rechtsprechungslinien sind auf ein pandemisches Szenario nur bedingt übertragbar. Während bisher singuläre Äußerungen im Mittelpunkt standen, geht es nunmehr um über Monate und Jahre andauernde Kommunikation. Das Gebot der Sachlichkeit könnte in einer solchen Situation zu modifizieren sein: Die fehlende Eilbedürftigkeit könnte sich dahin gehend auswirken, dass die Anforderungen an Sachlichkeit (und politische Neutralität) anders zu verstehen sind.

Darüber hinaus betrachtet das Forschungsprojekt die Frage, ob und wie staatliche Einrichtungen soziale Medien für eigene Kampagnen nutzen dürfen und wie Beiträge dort rechtskonform ausgestaltet werden können. Dazu entwickelt es ein Rechtsdesign, das klare Vorgaben für rechtssichere und effektive Kommunikation enthält.

## Fazit und Ausblick

Die ausgeführten Forschungsdesiderata verdeutlichen die Notwendigkeit eines interdisziplinären Forschungsverbundes aus Medien‑, Kommunikations‑, Verhaltens- und Rechtswissenschaft. Gefördert durch das Bundesministerium für Bildung und Forschung werden seit Herbst 2021 bis zum Jahr 2024 Erkenntnisse zum evidenzbasierten Einsatz multimodaler Kommunikation durch staatliche und mediale Institutionen sowie Social-Media-Akteur/-innen generiert.

Von diesen Daten profitieren nicht nur die Einzeldisziplinen, sondern auch die Gebiete der Risiko‑, Krisen‑, Gesundheits‑, Wissenschafts- und Sicherheitsforschung, deren Erkenntnisschnittmengen im Artikel skizziert wurden. Zudem werden über adäquate Formate der Wissenschaftskommunikation Verantwortliche und Beschäftigte in Behörden, Organisationen der Gesundheitssicherung und Medienhäusern erreicht. So findet bereits im Sommer 2023 eine Ausstellung im Berliner Museum für Kommunikation statt, die einen ersten Schritt darstellt, die entsprechenden Akteur/-innen für eine Integration multimodaler Ansätze in behördliche und mediale (Krisen‑)Kommunikations- und Informationsstrategien zu sensibilisieren.

## Footnotes

1 Nähere Informationen zum Forschungsverbund, zu Inhalten und Zielen sowie zu Neuigkeiten des Projektes finden sich unter: https://mirkkomm.de/ (abgerufen am: 11.01.2023).

2 U. a. www.rki.de/covid-19-steckbrief.

3 Debunking meint, Fehlinformationen zu entlarven und Maßnahmen zu entwickeln, um diesen entgegenzuwirken.

4 Erkenntnisse zu „risk literacy“ finden sich z. B. hier: https://www.hardingcenter.de/de.
